# Lessons from the War

**Published:** 1900-05-19

**Authors:** 


### LESSONS FROM THE WAR.

In his interesting address on the wounded in the present war, delivered before the Royal Medical and Chirurgical Society, Mr. Treves directed attention more particularly to three points: (1) The disproof offered by the experience gained in the present war of certain commonly accepted statements as to bullet wounds of bones; (2) the necessity for trephining in every case of gunshot injury of the skull; and (3) the lessons taught in regard to the treatment of gunshot wounds of the abdomen. In regard to the first of these points he picks out four prominent statements made in recent text-books which, he says, do not seem to be borne out by experience in the present war. The first is that the severity of the injury to bone decreases as the range increases; the second is that explosive effects are produced when a bone is hit at short range, such as 500 yards or under; the third is that gunshot fractures are nearly always oblique; and the fourth is that when a bone is fractured the exit wound is always larger than when this does not occur. In none of these does experience support the text-books. In regard to head injuries Mr. Treves seems to have arrived at the impression that the majority of those killed on the spot were shot through the head. As to those who lived to be brought in he says that no operations in the field did anything like so well as those done for head injuries, and he lays down the rule that it is not a question of choosing in which case to operate, but that one should "operate in every single instance of gunshot wound of the head." It is interesting to see that Mr. Watson Cheyne[1] has arrived at almost precisely the same conclusion. He says, "the experience of bullet wounds through the skull seems to show that a good rule to follow is to trephine immediately over the aperture of entrance, however trivial the injury apparently is." On opening up such a wound, he says, the inner table is found broken into fragments, which are driven into the brain, sometimes for a very considerable distance. Where the entrance and exit wounds are near together, even where the bullet has traversed the brain substance between them, he has found that the skull lying between the two openings, for about an inch in breadth, has been broken into small fragments. In many cases the symptoms were quite slight until, later, convulsive attacks supervened, or internal suppuration occurred. In regard to abdominal bullet wounds, if one puts on one side the cases in which recovery may take place without interference—a far larger class than might have been expected—and those in which there is no likelihood of operation done under the circumstances doing good, "the cases that are suited for abdominal section in the field are exceedingly few." In describing what these circumstances sometimes are, he says: "Think of the difficulties of operating on an abdominal case. . . . There was no water; at least, there was water, but it was not anything that we are accustomed to call water. It will be said, Why not boil it? But where is the fuel? There is nothing on the veld but grass. We are 26 miles from the railway. Some may say, Why not use methylated spirits? Can you carry that about in any quantity? Others may say, Filter the water. Well, I never could get anything like half a bottle of water filtered without having to take the filter to pieces to clean it, because it was found to be thickly coated with mud. . . . You cannot realise starting an abdominal section without towels. You may say, Have plenty of towels; but if we started with 600 towels, where are the laundries? There is the veld, and there is the Tugela; but the Tugela is covered with Boer rifles. What is to be done? You say, Well, take a thousand towels—that means 16 more oxen, and even then your towels are no good. It cannot be done. In the country you cannot manufacture water, and you cannot produce in the middle of the desert a complete laundry. . . Another thing which rendered abdominal surgery almost impossible was the trouble we had with flies. . . . One's hands were covered with flies as one operated; put them away as one might they were there again. If a piece of bowel came out it was covered with flies. We kept wisps going, and we tried all sorts of things, but you cannot keep the South African fly away." These being the conditions in which any operation had to be done, all the more interest attaches to a consideration of the sort of cases that got well when let alone. Although Mr. Treves said he would not trouble his audience with any "bullet stories," he did as a fact tell some very good ones, and the very fact that such wonderful recoveries do take place is another reason to make one chary of doing laparotomy if there is the slightest doubt about its absolute necessity. In many cases, he says, beyond the fact that the men were shot in the belly nothing occurred. "They were shot through the abdomen, and it was interesting to them to know that; but there was nothing else to notice. One of the cases was that of a bullet close to the umbilicus; the bullet came out by the spine of the second lumbar vertebra. What happened to the structures like the big blood-vessels I do not know. One bullet went in in the anterior part of the loin, and came out at the other loin, after having traversed the whole belly. One went in exactly over the stomach, and came out over the right loin; another went in at the tip of the eleventh rib, and came out at the axilla. In none of these cases did the men have any more than what would be called trifling symptoms, such as they would get from eating a green apple." Another case he mentioned was that of a man who was crawling up towards a ridge when he was struck by a bullet, which entered above the collar-bone in the neck, and came out on the inner side of the opposite thigh, "literally without producing any inconvenience or any symptom of any sort whatsoever, except that the man, being wounded, thought he had better take advantage of it and get the bread and other luxuries that we might have for the wounded."

[1] Brit. Med. Jour., May 12.

